# Artificial Intelligence to Address Cyberbullying, Harassment and Abuse: New Directions in the Midst of Complexity

**DOI:** 10.1007/s42380-022-00117-x

**Published:** 2022-02-25

**Authors:** Tijana Milosevic, Kathleen Van Royen, Brian Davis

**Affiliations:** 1 Anti-Bullying Centre and ADAPT SFI, Dublin City University, Lower Drumcondra Road Dublin 9, Co., Dublin, D09 DW93 Ireland; 2 University of Antwerp, Sint-Jacobstraat, 22000 Antwerpen, Belgium; 3 School of Computing and ADAPT SFI, Dublin City University, Glasnevin Campus, Room Number, Dublin, L2.26 Ireland

**Keywords:** Cyberbullying, Moderation, Abuse

## Abstract

This brief article serves as an introductory piece for the special issue “The Use of Artificial Intelligence (AI) to Address Online Bullying and Abuse.” It provides an overview of the state of the art with respect to the use of AI in addressing various types of online abuse and cyberbullying; current challenges for the field; and it emphasises the need for greater interdisciplinary collaboration on this topic. The article also summarises key contributions of the articles selected for the special issue.

## AI and Moderation

Much like offline abusive behaviours, online bullying, harassment and abuse continue to pose a significant problem for children and adults alike. Some evidence suggests that their prevalence has increased with the COVID-19 lockdowns (Keating et al., 2020; Lobe et al., 2021; Milosevic, Laffan, et al., 2021). While parents/guardians, schools/educators and the government have an important role to play in addressing all forms of bullying, online platforms such as social media, games and direct/private messaging, among others, are also key actors in this process, and they are struggling to find ways to more effectively moderate bullying behaviours (Gillespie, 2018; Milosevic, 2018). *Moderation* refers to examining content that is reported to online platforms for the purposes of assessing whether (a) it violates the platform’s policy and (b) is subject to eventual removal as such violative content. Abuse, harassment, cyberbullying and hate speech typically constitute breaches to platform policy.

The vastness of shared content on platforms makes it impossible to rely on human moderation only (not to mention the psychological damage that human moderation can entail for moderators because of the sensitivity and emotionally heavy nature of the content itself; see Roberts, 2019), and recent years have witnessed a steady increase in research efforts to find effective ways to leverage algorithmic techniques intended to help automate the process of moderation, such as natural language processing (NLP), machine and deep learning (from now on artificial intelligence or AI), to effectively address the problem (Gorwa et al., 2020; Gillespie et al., 2020; Vidgen & Derczynski, 2020). This would allow for a more effective triaging of content for human moderation, and it would also enable greater reliance on proactive moderation. Unlike reactive moderation, where a user reports a piece of content which is subsequently sent to moderation and processed in an automated fashion or alternatively investigated by humans, proactive moderation relies on the aforementioned techniques to automatically detect such instances of policy violation before they are reported by users. Some of the large platforms such as Facebook/Meta, Twitter and Google already publish the percentage of such cases that have been detected and actioned before they were reported. However, it is not always entirely clear as to what such actioning of content entails, and how it is done, and scholars, the media and governments alike have voiced concerns regarding the lack of transparency and accountability of online planforms. Recent activities of the Office of the *e*Safety Commissioner in Australia (Australian Government, n.d.), and legislative developments in Europe such as the Online Safety Bill in the UK (Gov.UK, 2021) and the Online Safety and Media Regulation Bill (OSMR) in Ireland (Government of Ireland, 2022), promise to deliver greater scrutiny by enabling the government to better examine company activity via auditing and implementation of codes of conduct by companies. The importance of understanding companies’ work will increase as they expand into virtual reality, robotics and with a greater use of wearable and other “smart” devices (i.e. internet-connected devices, such as toys, virtual assistants, “smart” home appliances).

## Why Are Abuse, Cyberbullying and Harassment Difficult to Moderate?

These phenomena involve not only overtly abusive texts, which tend to be easier to detect, but also irony and sarcasm, which can still be difficult to decipher via algorithmic learning approaches despite many recent advancements (Chia et al., 2021; Tommasel et al., 2018). Furthermore, abusive words such as “bitch” or expletives can be used in a playful or friendly manner, resulting in false positives. To add to that, abusive content can be multimodal, involving text, image and video, where only one component can be abusive or none of the components is abusive on their own but rather they are only abusive when considered as a whole (Kumar & Sachdeva, 2021). For example, consider a photo of a tombstone with a comment underneath “you belong here” (Facebook AI, 2020). Another issue is identifying exclusion in instances of bullying. A few years ago, for example, it came to our attention that a popular way for girls to bully each other on Instagram was to post photos of themselves and then tag the girl or girls who had not been invited, to show them that they are excluded (Davis, 2019). Automating the detection of such instances could result in false positives—for example, all those cases where someone was not in the photo perhaps because they could not attend the event but were tagged for fun and in fact inclusion. A whole other area is involving behavioural interactions (such as user interactions and temporal dimensions, likes, shares, replies, re-posting) and leveraging social network analytics to detect cyberbullying (Ge et al., 2021). Many of the classifiers are still able to only categorise content or behaviours as abusive or not abusive, for instance, based on hateful language or slurs, and they do not provide a more nuanced description of the type of abuse involved, or the severity of the case; or roles played by those involved (for examples of multi-class classifiers; see, e.g. Balakrishnan et al., 2019; Jacobs et al., 2020). Furthermore, for a case of abuse to be classified as “cyberbullying”, there still (according to widely used definitions) typically needs to be some level of repetition, intent to hurt and even power imbalance (although said definitions are under revision and tend to be intensely debated)^1^ and classifiers for the most part do not capture those (Cheng et al., 2020). Some classifiers attempt to identify bystanders, victims and the perpetrator (roles) (Emmery et al., 2021; Van Hee et al., 2015).

In our recent scoping study paper (Milosevic, Verma, et al., 2021), we outline some of the challenges to creating classifiers that are able to detect more instances of bullying. Among these is the lack of datasets that contain sufficient examples of a variety of cyberbullying content, and perhaps more importantly, datasets which have been annotated by experts and with annotation guidelines that have been developed in collaboration with social scientists. Such collaboration is incredibly important, especially given the growing interest among computational linguists, as well as machine and deep learning computational scientists into this area. But if technical scholars do not have sufficient social scientific understanding of the phenomena for which they are trying to create automated solutions, they may inadvertently replicate/permeate design flaws or biases into their models. Technical scholars’ solutions might involve ethical dimensions of which they might not be fully aware.

Recent attempts to build a classifier that would detect levels or severity of cyberbullying cases could provide a pertinent illustration of the issue in question (on severity of cyberbullying see Hinduja & Patchin, 2019; Palladino et al., 2017). Building a classifier that would be able to detect the severity of a cyberbullying incident is presumably driven by the need to facilitate more effective content moderation, especially for online platforms. This would allow, for example, the largely automated moderation systems to triage decisions as to which cases should be brought to the attention of human moderators first (i.e. prioritised for human moderation). Yet, the classification of cyberbullying cases or instances based on severity level that is to be fed into a state-of-the-art natural language processing architectures such as BERT^2^ has to undergo a systematic social scientific conceptualization before it is provided to computational scholars for further processing (Devlin et al., 2018; Lutkevich, n.d.). BERT stands for bidirectional encoder representations from transformers, and it is a deep learning framework designed by Google and made open source (freely available for redistribution and modification). BERT assists computers to correctly process the meaning of ambiguous words in text by learning simultaneously their surrounding context (Delvin et al., 2018). There needs to be a social scientific rationale rather than merely a linguistic one as to why certain cases are considered as “high” vs. “medium” and “low” severity type of abuse, harassment or cyberbullying. For example, if one was doing a study with data from Twitter and was to find that sexual and appearance-based abusive tweets contained similar profane words, this linguistic rationale would not be sufficient from a social scientific perspective to cluster these tweets into a category such as “high severity tweets” (Talpur & Sullivan, 2020). The logic behind categorising a tweet as “low” vs. “high” severity abuse, harassment or cyberbullying needs to be explicitly stipulated in order to be replicable in future studies.

Different annotators may have various understandings as to what a “severe threat” is: such category should be defined and operationalised with specific guidelines for annotators (Van Hee et al., 2015). For example, if cyberbullying cases^3^ that include profane words are to be classified as “high severity”, then what is the evidence that such assumption is based on? Is it because there is social scientific research-based evidence that shows that children or whoever the target group is, are more negatively impacted by communication that includes profane words? Or is there some other rationale for labelling the presence of a profane word as “high severity”? Secondly, if “appearance” and “sexual” attacks are then banded together into one category as “high severity” because they both include profanity words, and because “sexual harassment” was labelled by one social scientific study (based on American adult population) as a more severe form of harassment, while racial harassment^4^ and intelligence-based offences are categorised as “medium” and “low” severity respectively (Talpur & Sullivan, 2020), then the understanding which informed such classification would need to be made explicit and justified. Which social scientific sources demonstrate that appearance-based cyberbullying is considered as more severe or harmful (and “harm” is presumably operationalised as “hurt/hurtful”) than insults that attack someone’s intelligence? This will likely vary from individual to individual and may also be different among genders, age-groups and cultures. For example, some girls might be particularly sensitive to weight-related offences during adolescence, while some boys of the same age may not find insults that target their body mass to be equally hurtful. Having an explicit understanding of the social scientific evidence that informed the logic behind classification is essential in order to prevent unintended consequences such as having platforms classify cases that might have severe psychological impact on a child as “low severity” and therefore “low priority” cases. We should also consider that prioritising “severe threats” (however, this might be operationalised) over remarks that insult intelligence or embarrass can also have palpable psychological consequences for a child who is continuously bullied based on their intelligence but not physically threatened. Decisions around prioritisation can have profound consequences for users.

Prioritising moderation of bullying content based on “views” is another example (Bickert, 2020). If a company decides to more promptly address bullying cases that have been viewed by more people over those that have been viewed by fewer people, under the presumption that the former create greater harm, then the child who has been bullied by a small number of people and where such bullying is confined to a handful of children who viewed such content will be underprioritized, even if that situation might be time critical, i.e. the child urgently needs help. In fact, that very child might suffer intense damage from such a case.

## Our Intentions Behind This Issue

Our primary goal with this issue is to highlight the relevance of the field of Artificial Intelligence (AI) development to researchers who study bullying and various forms of abuse across social scientific disciplines and to emphasise the urgent need for greater collaboration and communication with the field of computational science for the reasons outlined above. We, therefore, wished to compile a multidisciplinary selection of articles that illustrate various uses of AI for the detection of different forms of online abuse from bullying to hate speech; how AI is being leveraged to design intervention and prevention measures; how it can inform social scientific work but also to emphasise its limitations (sometimes unintended and unexamined), flaws in design and how its implementation can further existing social inequalities, which is a widely researched and acknowledged issue (Gebru, 2019; Raji et al., 2020). This field is developing very fast and by the time this issue will have been published, the state of the art will have advanced even further, and we are aware of this limitation. The idea is to provide merely a sketch of a burgeoning research agenda that demands deeper social scientific scrutiny.

## Selected Articles

In *Bullying-related Tweets: A Qualitative Examination of Perpetrators, Targets and Helpers*, Dr. Karla Dhungana Sainju, Akosua Kuffour, Lisa Young and Niti Mishra illustrates how computational methods are being used to collect and process data from social media that can assist in overcoming self-report biases of traditional social scientific methods such as surveys. A significant number of tweets containing bullying traces were collected, classified as pertaining to the victim, perpetrator or bystander, and examined via qualitative analysis as to the characteristics of the content of the tweets pertaining to each. The findings provide important insights for social scientists into the nature of cyberbullying incidents and behaviours of each actor in the process; they can also be leveraged for the development of classifiers for more effective detection of cyberbullying incidents and the identification of bystanders whose help can be solicited when designing interventions to assist victims.

In *Think Twice to be Nice: A User Experience Study on a Reflective Interface to Reduce Cyber Harassment on Social Networking Sites*, Dr. Kathleen Van Royen, Dr. Karolien Poels, Prof. Heidi Vandebosch and Dr. Bieke Zaman provide further insight into the “reflective messages”, an interface design which uses AI to prevent users from posting harassing content by prompting them to take time to think whether they really want to post it. The article builds on previous research into the effectiveness of this strategy by specifically examining how adolescents appreciate these reflective messages and evaluates its pragmatic design and hedonic qualities. The study demonstrates that adolescents’ evaluations are positive; however, strategies to avoid user fatigue in the long-term are needed. The article provides a useful reflection on the need to ensure that such interventions are user-friendly and desirable for young users, which can help inform their implementation on online platforms.

In *Curating Cyberbullying Datasets: A Human AI-Collaborative Approach*, computational scholars Dr. Christopher E. Gomez, Dr. Marcelo O. Sztainberg and Dr. Rachel E. Trana, illustrate a novel approach to annotation of cyberbullying data, which seeks to overcome biases inherent in human annotation such as inaccuracies due to cultural and language barriers and subjectivity. They apply AI algorithms inspired by the human brain (neural networks) to provide insights into which data is consistently labelled as bullying vs. non-bullying and discuss how their method could improve the accuracy of annotations in similar datasets.

In *Harnessing the Power of Interdisciplinary Research with Psychology-informed Cyberbullying Detection Models*, Dr. Deborah Hall, Dr. Yasin Silva, Brittany Wheeler, Lu Cheng and Kathleen Baumel explain the ways in which machine learning models for detecting cyberbullying could inform a more nuanced understanding of the psychological aspects of cyberbullying. The article further highlights how machine learning approaches can result in practical implications for families, clinicians and overall prevention and intervention.

Prof. Eugenia Siapera’s article *AI Content Moderation, Racism and (De)coloniality* provides a much-needed critical reflection on AI-based content moderation with the focus on racist hate speech. By scrutinising the publicly available information on how such moderation is designed and implemented, she puts forth a compelling argument that platforms’ approach to hate speech, which disregards the experience of racialised people and expropriates their labour with little or no compensation, in fact reproduces rather than eradicates racism.

*A Mobile-based System for Preventing Online Abuse and Cyberbullying*, by Dr. Semiu Salawu, introduces BullStop, a new cyberbullying detection app, which was trained on Twitter data. Readers may have encountered apps that have been designed over the past few years that rely on AI (in an attempt) to assist children and adults alike with cyberbullying by blocking abusive messages, by deploying the technique of reflective messaging discussed above to deter perpetration or by providing assistance and educational advice to those involved in bullying cases. Dr. Salawu’s article describes the process of designing such an app and how it sought to overcome the computational challenges normally encountered in this process, as well as how it leveraged input on the app design from users via social scientific methods.

We thank the authors for sharing their work, and we hope that our readers will find the compilation of articles engaging that it will stimulate future multidisciplinary collaborations which will foster critical reflections and scrutiny, and in so doing, benefit society.

## Footnotes

1 https://events.unesco.org/event?id=24578225&lang=1033

2 https://towardsdatascience.com/bert-explained-state-of-the-art-language-model-for-nlp-f8b21a9b6270

3 We need to be clear as to what is defined by “a case”, is one tweet/post enough for something to be classified as “cyberbullying” or does the criterion of repetition, which remains inherent in cyberbullying definitions, need to be upheld as well?

4 Racial harassment which strictly speaking may fall under hate speech rather than cyberbullying.
